# Development of cryptococcal immune reconstitution inflammatory syndrome 41 months after the initiation of antiretroviral therapy in an AIDS patient

**DOI:** 10.1186/s12981-015-0075-6

**Published:** 2015-09-30

**Authors:** Hideki Hashimoto, Shuji Hatakeyama, Hiroshi Yotsuyanagi

**Affiliations:** Department of Infectious Diseases, The University of Tokyo Hospital, 7-3-1 Hongo, Bunkyo-ku, Tokyo, 113-8655 Japan; Division of General Internal Medicine/Division of Infectious Diseases, Jichi Medical University Hospital, 3311-1 Yakushiji, Shimotsuke-shi, Tochigi, 329-0498 Japan

**Keywords:** Cryptococcus, HIV infection, Immune reconstitution inflammatory syndrome, AIDS, Antiretroviral therapy

## Abstract

Cryptococcal meningitis is one of the most lethal fungal infections in patients with acquired immune deficiency syndrome (AIDS). The incidence of and mortality from cryptococcal meningitis have markedly decreased since the introduction of combination antiretroviral therapy (cART). However, despite its benefits, the initiation of cART results in immune reconstitution inflammatory syndrome (IRIS) in some patients. Although IRIS is occasionally difficult to distinguish from relapse or treatment failure, the distinction is important because IRIS requires a different treatment. Here, we present the case of a patient with AIDS who developed symptoms of cryptococcal IRIS 41 months after starting cART. To the best of our knowledge, the time between cART initiation and the onset of cryptococcal IRIS in this patient is the longest that has been reported in the literature.

## Background

Cryptococcal meningitis is one of the most lethal fungal infections in patients with acquired immune deficiency syndrome (AIDS) [1]. As an opportunistic infection, it may develop in patients whose CD4 cell counts are less than 100 cells/μL. Before the introduction of combination antiretroviral therapy (cART), cryptococcal meningitis developed in 6–10 % of AIDS patients [1]. However, since the introduction of cART, the incidence of and mortality from cryptococcal meningitis and other opportunistic infections have decreased markedly in AIDS patients [2].

Some patients experience immune reconstitution inflammatory syndrome (IRIS) after the initiation of cART. IRIS is thought to occur when cART causes immune system recovery and thereby elicits paradoxical inflammatory responses to either previously diagnosed and treated or subclinical infections [3].

IRIS has been described in association with multiple opportunistic pathogens, such as *Mycobacterium avium* complex*, Mycobacterium tuberculosis*, *Cryptococcus neoformans*, cytomegalovirus, and hepatitis viruses. Although IRIS was initially described in human immunodeficiency virus (HIV)-infected patients who had started cART, it is now known to occur in other settings as well, such as in organ transplantation, patients after cessation of tumor necrosis factor (TNF)-α inhibitors, and pregnancy [4–6]. Cryptococcal IRIS most frequently presents as recurrent meningitis; however, it may manifest as intracranial space-occupying lesions and non-central nervous system presentations (e.g., lymphadenitis, pneumonitis, and ophthalmologic complications) [7]. Previous studies have reported that cryptococcal IRIS develops in 8–45 % of HIV-infected patients with cryptococcosis who responded to cART, despite the use of antifungal therapy. The mortality rate among these patients ranges from 8 to 30 % [7–10].

Generally, IRIS occurs within a median of 12 weeks after starting cART [11]. Similarly, most cases of cryptococcal IRIS have been shown to develop within 12 months after the initiation of cART, with median intervals ranging from 1 to 10 months [7]. However, IRIS can also occur outside of the usual range. For example, cryptococcal IRIS has been reported as early as 4 days and as late as 37 months after starting cART [7, 8, 12].

In the following section, we present the case of an AIDS patient who developed symptoms of cryptococcal IRIS 41 months after starting cART. To the best of our knowledge, the time between cART initiation and the onset of cryptococcal IRIS in this patient is the longest that has been reported in the literature.

## Case presentation

In December 2009, a 59-year-old man developed *Pneumocystis jirovecii* pneumonia (PCP) and was diagnosed as having HIV-1 infection. His CD4 T cell count was 44 cells/μL and his HIV RNA load was 16,000 copies/mL at diagnosis. He also had chronic hepatitis B. Two weeks after completing the treatment for PCP using adjunctive corticosteroids, he was admitted to our hospital again after presenting with a gradually worsening headache and fever. On admission, he had no neck stiffness, photophobia, or focal neurological abnormality. A computed tomography (CT) scan of the head did not show any pathological findings. A lumbar puncture revealed an opening pressure of 350 mm H_2_O, cell counts of 7/µL (100 % lymphocytes), protein levels of 29 mg/dL, and glucose levels of 40 mg/dL. Yeasts were seen on a cerebrospinal fluid (CSF) smear. Both the CSF and blood cultures subsequently became positive for *C. neoformans* with minimum inhibitory concentrations of 1.0 µg/mL for amphotericin B, 4.0 µg/mL for fluconazole (FLCZ), and 4.0 µg/mL for flucytosine (5-FC). The patient’s initial blood and CSF cryptococcal antigen titers were 1:16,384 and 1:4096, respectively. A chest X-ray film showed no abnormalities; however, white perivascular infiltrates that were compatible with fungal retinitis/endophthalmitis were observed in both ocular fundi. The patient was therefore diagnosed with disseminated cryptococcosis with meningitis, retinitis, and cryptococcemia. He was treated with amphotericin B lipid complex (L-AmB) 200 mg/day and 5-FC 6000 mg/day (which was decreased to 4000 mg/day because of the adverse effect of pancytopenia) for 2 weeks, followed by L-AmB 200 mg/day and FLCZ 400 mg/day for another 2 weeks. The initial treatment was followed by maintenance therapy (FLCZ 200 mg/day). Fungal clearance was observed in the CSF obtained 15 days after starting antifungal therapy. Initial and subsequent CSF findings are shown in Table 1.

**Table 1:** Initial and subsequent cerebrospinal fluid findings

	Feb 2010 (Day 1 of therapy)	Feb 2010 (Day 8 of therapy)	March 2010 (Day 15 of therapy)	March 2010 (Day 43 of therapy)	Sep 2013
Cell counts (/µL)	7	10	4	1	9
Rate of mononuclear cells (%)	100	100	NT	NT	100
Protein (mg/dL)	29	33	31	29	82
Glucose (mg/dL)	40	49	41	45	58
CSF-to-serum glucose ratio	0.41	0.32	0.36	0.48	0.59
Fungal smear	Positive	Positive	Positive	Negative	Negative
Fungal culture	Positive	Positive	Negative	Negative	Negative
Cryptococcal antigen titer	1:4096	NT	1:2048	NT	1:4

*CSF* cerebrospinal fluid, *NT* not tested

In March 2010, 5 weeks after the initiation of antifungal therapy for cryptococcosis, cART with tenofovir/emtricitabine plus lopinavir/ritonavir (LPV/r) was started. In May 2010, raltegravir replaced LPV/r due to the hepatotoxicity associated with LPV/r. The HIV RNA load in the blood became undetectable within 3 months after starting cART. His CD4 T-cell count recovered gradually to about 200 cells/μL without recurrence of PCP or cryptococcosis. To provide maintenance therapy for cryptococcal meningitis, FLCZ was continued alongside cART.

**Fig. 1:**
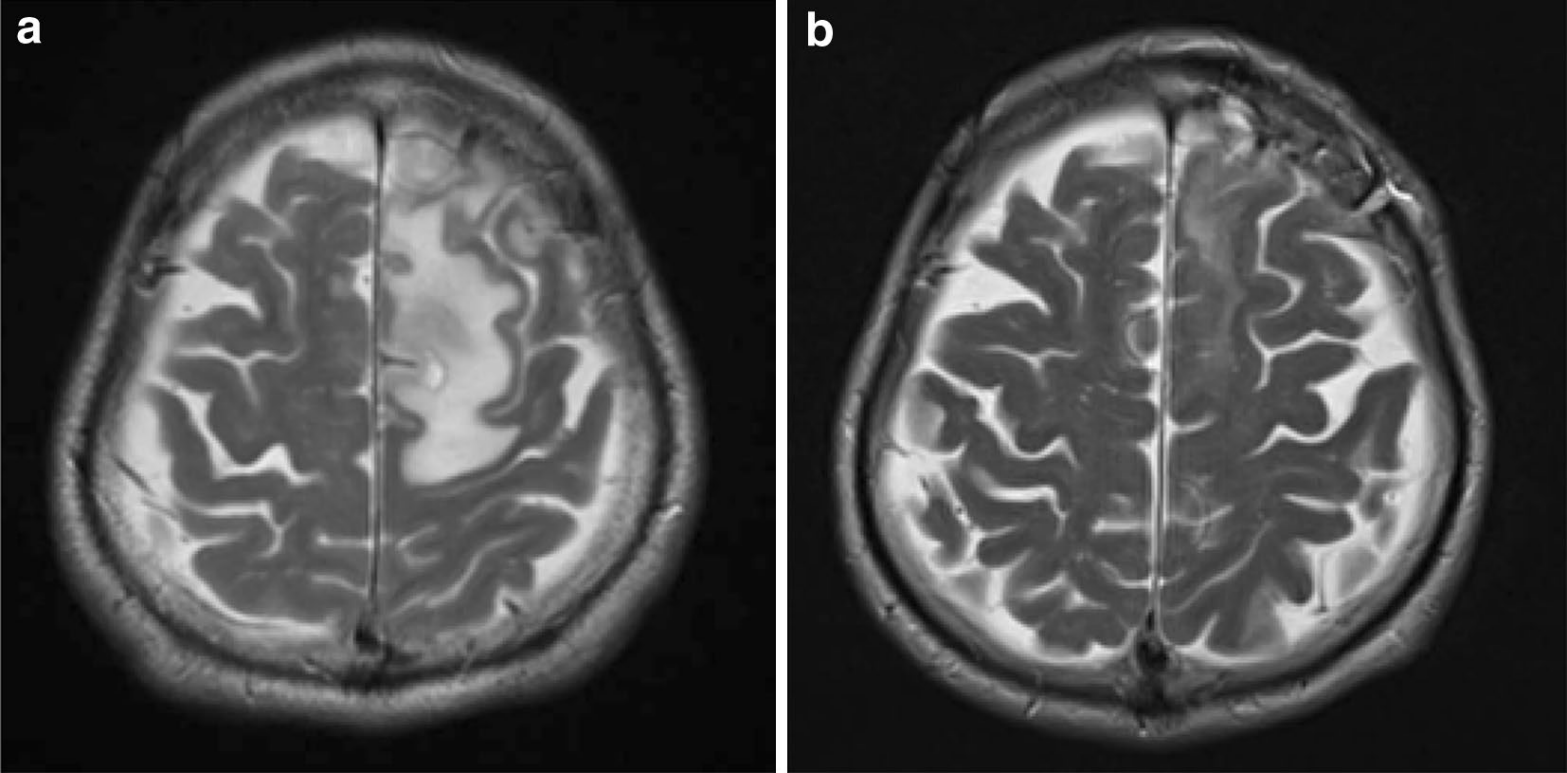
MRI scans of the brain on admission and 3 weeks after the initiation of therapy. An MRI scan on admission **a** showed high signal intensity on T2-weighted images in the white matter of the left temporal and right frontal lobes. Obvious mass effects were not observed. Contrast enhancement was seen along the brain surface and cerebral sulci; however, little enhancement of the brain parenchyma was detected. A follow-up brain MRI scan obtained 3 weeks after the initiation of antifungal and steroid therapy **b** showed overall improvement with a marked decrease in the size of the white matter lesions

**Fig. 2:**
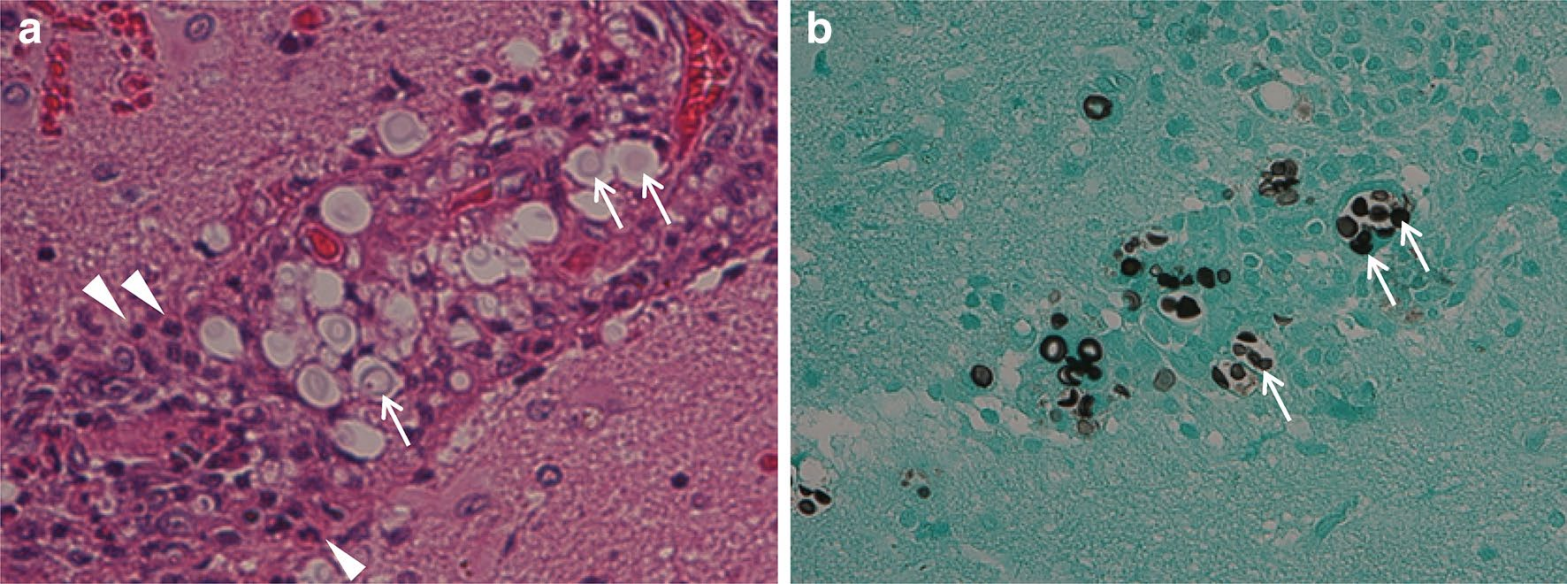
The pathological examination of the brain biopsy. The pathological examination of the brain biopsy of the left frontal lobe revealed inflammatory cells, such as polymorphonuclear cells (*white arrowheads*), and many rounded yeast-like cells (*white arrows*) in the subarachnoid cavity and periarteriolar spaces. Fungal structures compatible with *Cryptococcus* species were observed on periodic acid-Schiff (**a**) and Grocott’s (**b**) staining (magnification ×100)

In mid-August 2013, after 41 months of cART, the patient had a mild headache accompanied by mild nausea. Approximately 1 week later, he presented with a generalized tonic–clonic seizure featuring worsening paralysis of the right leg. He was still receiving cART and FLCZ maintenance therapy for cryptococcosis, with good adherence. His CD4 T-cell count was 193 cells/μL, and his HIV RNA load was undetectable upon admission. A CT scan of the head revealed low-density areas in the subcortical region and deep white matter with surrounding edema in the left frontal and right temporal lobes. A brain magnetic resonance imaging (MRI) scan (Fig. 1a) showed high signal intensity on T2-weighted images without obvious mass effect. Contrast-enhanced MRI showed enhancement along the brain surface and cerebral sulcus, but the parenchyma showed little enhancement. A lumbar puncture was performed and showed clear CSF (9 white blood cells/μL) as well as normal glucose and protein levels (Table 1). India ink stains and cultures of the CSF sample were negative. Blood and CSF cryptococcal antigen tests were both positive; however, titers had decreased to 1:64 and 1:4, respectively, in comparison with the titers that had been obtained in 2010 (Table 1). Because we did not perform blood or CSF cryptococcal antigen tests between March 2010 and August 2013, it could not be determined whether the blood and CSF cryptococcal antigen titers had ever fallen below 1:64 and 1:4, respectively. Polymerase chain reaction tests of the CSF for herpes simplex virus, varicella zoster virus, Epstein-Barr virus, and JC virus were all negative. A diagnostic brain biopsy of the left frontal lobe was therefore performed. The pathological examination revealed inflammatory cells and many rounded fungi that were detected on periodic acid-Schiff and Grocott stains in the subarachnoid cavity and periarteriolar spaces (Fig. 2). These findings were compatible with cryptococcal meningoencephalitis with white matter edema. Cultures of the brain tissue specimen were negative for *Cryptococcus* species and other fungal and bacterial pathogens. Thus, the patient was diagnosed with cryptococcal meningoencephalitis (mainly involving the meninges) associated with IRIS.

He was treated again with L-AmB 200 mg/day and 5-FC 3000 mg/day in addition to corticosteroid adjunctive therapy [prednisolone 50 mg (1 mg/kg) daily] for 2 weeks. This was followed by maintenance therapy with FLCZ 300 mg/day (which was adjusted according to renal function). The prednisolone dose was gradually tapered over 6 months. Discontinuation of antiretroviral therapy was not necessary during the course of the disease. His headache and right leg weakness resolved quickly, and the follow-up brain MRI scan obtained 3 weeks after the start of therapy showed an overall improvement with a marked decrease in the size of the white matter lesions (Fig. 1b).

## Discussion

Recently, it has been recognized that cryptococcal IRIS presents in two distinct modes. Accordingly, the International Network for the Study of HIV-Associated IRIS (INSHI) proposed two clinical case definitions of cryptococcal IRIS among HIV-infected individuals. The first, paradoxical cryptococcal IRIS, presents as a worsening or recurring disease of previously recognized and treated cryptococcosis in the same or new anatomical sites, despite microbiological evidence suggesting that antifungal treatment has been effective. The second, ART-associated cryptococcosis, is defined as new-onset cryptococcosis that occurs or is unmasked after starting cART [7]. Although there is no universally accepted definition of IRIS, our patient met the criteria for paradoxical cryptococcal IRIS according to the INSHI definitions.

Although the timing of onset appears to be quite variable, cryptococcal IRIS usually develops within 12 months after initiating cART and almost all cases occur within 3 years [8]. The INSHI cutoff for the development of cryptococcal IRIS symptoms is 12 months after the initiation of cART; however, the network acknowledges that later onset of cryptococcal IRIS can occasionally occur [7]. Our patient developed symptoms of cryptococcal IRIS 41 months after starting cART, which is an extremely late onset.

In terms of its clinical presentation, cryptococcal meningitis associated with IRIS may sometimes be difficult to distinguish from relapses [13]; however, negative culture results and good FLCZ compliance (as secondary prophylaxis) may help to distinguish between the two [7, 14]. Musubire et al. have described features that are useful for distinguishing paradoxical IRIS from potential alternative diagnoses [14]. Our patient received cART for more than 3 years and showed (1) a gradual increase in CD4 T-cell counts, reaching almost 200/μL; (2) suppressed plasma HIV RNA levels that remained below the detection limit for more than 3 years; (3) good cART and FLCZ maintenance therapy adherence; and (4) negative brain tissue culture results and very low cryptococcal antigen titers in the CSF. These findings indicated cryptococcal IRIS rather than reinfection or relapse. Because nonviable yeasts can occasionally linger in the central nervous system (CNS), positive fungal staining with negative fungal cultures in the CNS samples is not considered to be enough evidence to diagnose relapse [14]. However, it should be taken into consideration that a negative cryptococcal culture is not an absolute requirement for the diagnosis of cryptococcal IRIS. For example, spinal fluid may be sterile when a patient is treated with amphotericin B or high-dose FLCZ [15]. In addition, cryptococcal antigen titers are considered to be unreliable for differentiating cryptococcal IRIS from the early phase of relapse [14].

It is important to distinguish relapse from late-onset cryptococcal IRIS because they require different types of treatment. Cryptococcal relapse may require re-induction antifungal therapy [14], while cryptococcal IRIS may require anti-inflammatory drugs, such as nonsteroidal anti-inflammatory drugs or corticosteroids [7, 15].

Several retrospective studies have reported the following risk factors for developing cryptococcal IRIS: higher pre-cART HIV RNA load, lower CD4 cell counts with a greater CD4 cell count increase in the first 6 months of cART, and a higher organism or antigen burden at baseline and at cART initiation [8, 10, 11]. Paucities of neutrophils, proteins, and cytokines (including TNF-α and interleukin-2, 6, 8, and 17) in the CSF have also been suggested to be risk factors for developing cryptococcal IRIS [16]. Although the timing of the start of cART following cryptococcal meningitis may be a predictor for the development of cryptococcal IRIS, the optimal timing of cART has yet to be determined and should be individualized [10].

Cryptococcal meningitis associated with IRIS is a potentially fatal disease with a mortality rate that is twice as high as that of cryptococcal meningitis in ART-naïve HIV-infected patients [17]. Given that increased intracranial pressure (ICP) (higher than 25 cm H_2_O) is often reported in patients with cryptococcal meningitis associated with IRIS, repeated lumbar punctures need to be performed to reduce the ICP in two-thirds of the cases [18]. Our patient was administered corticosteroids, which have sometimes been used previously for patients with serious conditions, such as extremely high ICP or focal neurologic deficits. Some experts use prednisone (0.5–1 mg/kg, daily) or an equivalent, whereas others may use a higher dose of dexamethasone that is then tapered over 2–6 weeks [19]. Although there is some evidence suggesting the need to discontinue cART in very severe cases, there is no need to discontinue cART in most non-severe cases [20].

## Conclusion

We have reported the case of a patient with AIDS who developed symptoms of cryptococcal IRIS 41 months after starting cART. Although cryptococcal IRIS usually develops within 1 year after starting cART, it can also occur much later. To the best of our knowledge, the time between cART initiation and the onset of cryptococcal IRIS in this patient is the longest that has been reported in the literature. It is necessary to carefully distinguish cryptococcal IRIS from relapse in such cases; although cryptococcal IRIS and relapse may have similar symptoms, each requires different treatments.

## Abbreviations

AIDS: acquired immunodeficiency syndrome
cART: combination antiretroviral therapy
IRIS: immune reconstitution inflammatory syndrome
HIV: human immunodeficiency virus
TNF: tumor necrosis factor
PCP: *Pneumocystis jirovecii* pneumonia
CT: computed tomography
CSF: cerebrospinal fluid
FLCZ: fluconazole
5-FC: flucytosine
L-AmB: amphotericin B lipid complex
LPV/r: lopinavir/ritonavir
MRI: magnetic resonance imaging
INSHI: International Network for the Study of HIV-Associated IRIS
CNS: central nervous system
ICP: intracranial pressure
